# Supplementary material for: The Potential Impact of Different Taxation Scenarios towards Sugar-Sweetened Beverages on Overweight and Obesity in Brazil: A Modeling Study
Source: Nutrients. 2022 Dec 4;14(23):5163. doi: 10.3390/nu14235163 (PMC9737639; doi:10.3390/nu14235163)
Supplement: Supplementary file 1 [file nutrients-14-05163-s001.zip › nutrients-2052365-supplementary.pdf]

Supplementary

Table S1. Estimate of the mean change in calorie intake (kcal/person/day) in the 3 taxation scenarios*, according to sex and age groups.

Sex	Age groups (years)	20% Tax Mean (95% CI)	25% Tax Mean (95% CI)	30% Tax Mean (95% CI)
**Male**	**20 to 29**	-10.5 (-12.9;-8.0)	-13.1 (-16.2;-10.0)	-15.7 (-19.4;-12.0)
	**30 to 39**	-7.5 (-9.9;5.0)	-9.4 (-12.4;-6.3)	-11.2 (-14.9;-7.6)
	**40 to 49**	-3.6 (-5.4;-1.8)	-4.5 (-6.8;-2.2)	-5.4 (-8.1;-2.6)
	**50 to 59**	0.3 (-0.9;1.6)	0.4 (-1.2;2.1)	0.5 (-1.5;2.5)
	**60 to 69**	1.5 (-0.2;3.1)	1.8 (-0.2;4.0)	2.2 (-0.3;4.7)
	**≥70**	3.3 (0.8;5.8)	4.2 (1.0;7.3)	5.0 (1.3;8.8)
	**All men**	-4.1 (-5.1;-3.1)	-5.1 (-6.4;-3.9)	-6.2 (-7.6;-4.7)
**Female**	**20 to 29**	-3.2 (-4.9;-1.4)	-3.9 (-6.2;-1.7)	-4.7 (-7.4;-2.1)
	**30 to 39**	-0.9 (-2.2;0.2)	-1.2 (-2.7;0.3)	-1.5 (-3.2;0.3)
	**40 to 49**	0.6 (-0.4;1.6)	0.7 (-0.6;2.0)	0.9 (-0.7;2.4)
	**50 to 59**	1.4 (-0.3;3.2)	1.8 (-0.4;4.0)	2.2 (-0.4;4.8)
	**60 to 69**	5.0 (3.8;6.2)	6.2 (4.7;7.7)	7.4 (5.6;9.3)

	**≥70**	6.8 (5.5;7.9)	8.5 (7.1;9.8)	10.2 (8.5;11.8)
	**All women**	0.9 (0.2;1.5)	1.1 (0.2;1.9)	1.3 (0.3;2.3)
**Overall**		-1.5 (-2.1;-0.8)	-1.9 (-2.7;-1.0)	-2.2 (-3.2;-1.2)

*****only sugar-sweetened soft drinks

Table S2 – Estimate of the mean variation of BMI (kg/m^2) in the 3 taxation scenarios*, according to sex and age groups.

		**Mean change in BMI in kg/m^2 (95% CI)**		
**Sex**	**Age groups**	**Tax 20%**	**Tax 25%**	**Tax 30%**
**Male**	**20-29**	-0.146 (-0.146;-0.145)	-0.182 (-0.183;-0.181)	-0.218 (-0.219;-0.217)
	**30-39**	-0.104 (-0.105;-0.104)	-0.131 (-0.131;-0.131)	-0.156 (-0.156;-0.155)
	**40-49**	-0.051 (-0.051;-0.051)	-0.064 (-0.064;-0.064)	-0.077 (-0.077;-0.076)
	**50-59**	0.004 (0.004;0.004)	0.006 (0.006;0.006)	0.007 (0.007;0.007)
	**60-69**	0.022 (0.022;0.022)	0.026 (0.026;0.026)	0.032 (0.032;0.032)
	**≥70**	0.049 (0.049;0.049)	0.062 (0.062;0.063)	0.074 (0.074;0.074)
	**All men**	-0.054 (-0.056;-0.053)	-0.068 (-0.070;-0.066)	-0.081 (-0.083;-0.079)
**Female**	**20-29**	-0.052 (-0.052;-0.052)	-0.063 (-0.063;-0.063)	-0.076 (-0.076;-0.076)
	**30-39**	-0.015 (-0.015;-0.015)	-0.019 (-0.020;-0.019)	-0.024 (-0.024;-0.024)
	**40-49**	0.010 (0.010;0.010)	0.011 (0.011;0.011)	0.015 (0.015;0.015)
	**50-59**	0.023 (0.023;0.023)	0.030 (0.030;0.030)	0.036 (0.036;0.036)
	**60-69**	0.084 (0.084;0.084)	0.104 (0.104;0.104)	0.124 (0.124;0.125)
	**≥70**	0.117 (0.116;0.117)	0.146 (0.146;0.147)	0.175 (0.175;0.176)
	**All women**	0.018 (0.017;0.019)	0.023 (0.021;0.024)	0.027 (0.026;0.028)
**Overall**		**-0.016 (-0.017;-0.015)**	**-0.020 (-0.021;-0.019)**	**-0.024 (-0.026;-0.023)**

*****only sugar-sweetened soft drinks

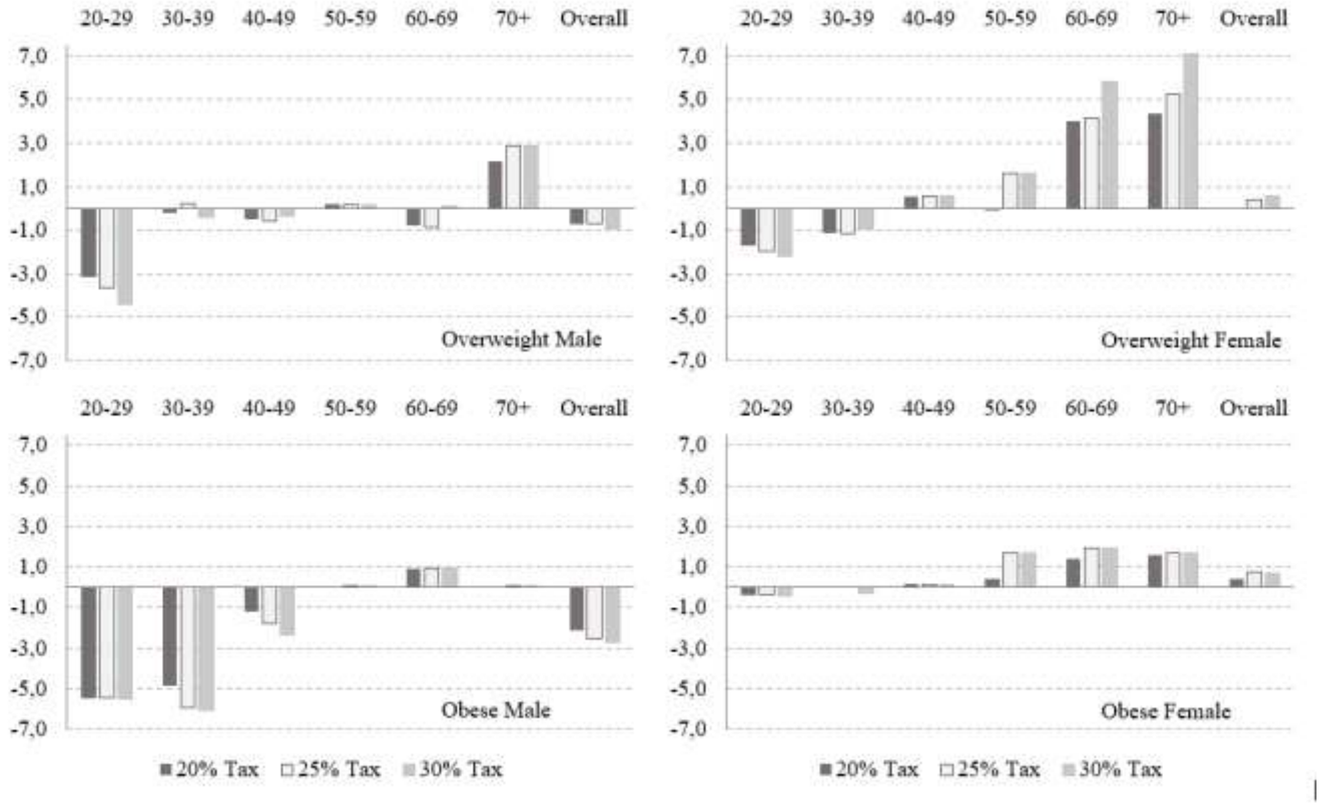

Figure S1. Percentage change in overweight and obesity prevalence (relative change in percent), by age and sex as a result of a 20, 25 and 30% sugar-sweetened soft drinks tax.

Table S3- Cross- and own-price elasticity according to beverage category.

**Group of beverages**	**Beverages**	Soft drink	Milk-based sweetened beverages	Sports and energy drinks	Other sugar-sweetened beverages	Fruit juice	Milk	Coffee and tea	Water
Soft drinks	Coke; Pepsi; lemon soda; apple soda.	-1.3634	0.2308	-0.0071	0.2302	0.0716	0.3574	0.3739	0.1174
Milk-based sweetened beverages	Dairy beverage; chocolate milk in a bottle or carton; milk shake; soy milk; light flavored milk; diet chocolate milk.	0.3700	-1.7481	-0.0042	0.1631	0.1608	0.3975	0.5611	0.0955
Sports and energy drinks	Sports drink; energy drink.	-0.4754	-0.1730	-0.2031	-0.1016	0.1993	0.1648	0.2871	0.1236
Other sugar-sweetened beverages	Fruit or vegetable juice in a carton; fruit syrup; powdered drink; instant cappuccino coffee; light mate tea.	0.4698	0.2052	-0.0031	-1.7305	0.0560	0.3913	0.5063	0.0998
Fruit juice	Orange juice; coconut water; fruit pulp; cane broth	0.2837	0.4031	0.0121	0.1130	-2.0308	1.2149	0.1731	-0.1110
Milk	Wholemeal cow's milk; skimmed pasteurized cow's milk; goat milk; fresh	0.1690	0.1165	0.0012	0.0912	0.1419	-0.8854	0.3301	0.0349

	milk; wholemeal milk powder								
Coffee and tea	Powder coffee; ground coffee; organic mate tea; mut herb	0.2944	0.2753	0.0034	0.1973	0.0338	0.5526	-1.4596	0.0854
Water	Mineral water; purified water; potable water	0.5556	0.2806	0.0088	0.2326	-0.1304	0.3494	0.5115	-1.8246

Source: FIPE (2020).

Table S4. Baseline data for mean body mass index (kg/m^2) according to age group and categories of body mass index, by sex. PNS, 2019.

	Male		Female	
Age groups (years)	n	Weighted* Mean BMI (95% CI)	n	Weighted* Mean BMI (95% CI)
20 to 29	6,335	25.2 (25.0 – 25.4)	6,626	25.3 (25.1 – 25.6)
30 to 39	8,796	27.2 (27.0 – 27.4)	9,039	26.9 (26.6 – 27.1)
40 to 49	7,958	27.3 (27.1 – 27.5)	8,586	27.4 (27.1 – 27.6)
50 to 59	7,394	27.1 (26.9 – 27.2)	8,255	27.4 (27.2 – 27.5)
60 to 69	5,802	26.6 (26.5 – 26.8)	6,752	27.2 (27.0 – 27.4)
≥70	4,391	25.8 (25.6 – 26.0)	5,781	26.2 (26.0 – 26.5)
Categories of BMI	n	Weighted* % (95% CI)	n	Weighted* % (95% CI)
<18.5 kg/m^2	564	1.3 (1.1 – 1.6)	1,101	2.4 (2.1 – 2.6)
18.5 – 24.9 kg/m^2	15,892	37.7 (36.9 – 38.5)	17,684	39.4 (38.6 – 40.2)
25 – 29.9 kg/m^2	16,612	41.1 (40.2 – 42.0)	15,964	34.7 (33.9 – 35.5)
≥30.0 kg/m^2	7,608	19.8 (18.9 – 20.7)	10,290	23.6 (22.8 – 24.5)

* Values are weighted for sample design

BMI: body mass index; 95% CI: 95% confidence interval

Table S5 – Parameters used in simulation model.

Parameters	Reference
Tax	20%, 25%, 30%
Pass on rate	100%
Cross- and own-price elasticities for beverages categories	Fipe, 2020[a]
Beverage consumption (baseline)	POF 2017-2018[b]
Energy balance	Hall et al. (2011)[c]
Anthropometric data (baseline)	PNS 2019[d]

[a]Fundação Instituto de Pesquisas Econômicas – FIPE (2020) Impactos sistêmicos das mudanças no padrão de consumo de bebidas açucaradas, adoçadas ou não, devido aos diferentes cenários de tributação: relatório final – atualização POF 2017/2018. São Paulo.

[b]Instituto Brasileiro de Geografia e Estatística (2020) Pesquisa de orçamentos familiares 2017-2018: análise do consumo alimentar pessoal no Brasil /IBGE, Coordenação de Trabalho e Rendimento. - Rio de Janeiro: IBGE. 120 p.

[c]Hall KD, Sacks G, Chandramohan D *et al.* (2011) Quantification of the effect of energy imbalance on bodyweight. Lancet **378**, 826–837

[d]Pesquisa Nacional de Saúde. 2019: atenção primária à saúde e informações antropométricas: Brasil / IBGE, Coordenação de Trabalho e Rendimento. - Rio de Janeiro: IBGE, 2020.

Table S6. Mean daily consumption in mL and 95% CI of SSBs in the scenario without taxation by category, sex and age groups, Brazil, POF 2017-2018.

Sex	Age groups (Years)	Soft drink	Milk-based sweetened beverages	Sports and energy drinks	Other sugar-sweetened beverages
		mL	mL	mL	mL
**Male**	**20-29**	219 (196, 242)	38 (29, 46)	2 (1, 4)	44 (35, 53)
	**30-39**	180 (156, 204)	17 (11, 23)	2 (0, 5)	48 (37, 58)
	**40-49**	139 (121, 157)	9 (6, 12)	1 (0, 1)	42 (31, 53)
	**50-59**	96 (83, 108)	9 (6, 13)	0 (0, 0)	34 (26, 41)
	**60-69**	79 (62, 96)	10 (4, 15)	-	27 (20, 34)
	**≥70**	71 (47, 95)	13 (5, 15)	-	37 (22, 52)
	**All men**	145 (135, 154)	17 (15, 20)	1 (1,2)	40 (36, 45)
**Female**	**20-29**	144 (129, 159)	42 (33, 50)	0 (0, 1)	44 (35, 52)
	**30-39**	109 (98, 120)	188 (14, 22)	1 (0, 1)	42 (34, 50)
	**40-49**	84 (75, 93)	11 (8, 22)	2 (0, 3)	28 (23, 33)
	**50-59**	84 (66, 102)	10 (7, 14)	0 (0, 1)	34 (29, 40)
	**60-69**	52 (43, 61)	13 (6, 19)	-	39 (31, 47)
	**≥70**	37 (29, 46)	11 (7, 15)	-	38 (29, 46)
	**All women**	91 (85, 97)	19 (16, 21)	1 (0, 1)	38 (34, 41)
**Overall**	176 mL (169, 184)	116 (110, 122)	18 (16, 20)	1 (0, 1)	36 (33, 40)

(-) There was no consumption.

Table S7 – Mean daily consumption in mL and 95% CI of non-sweetened beverages in the scenario without taxation by category, sex and age groups, Brazil, POF 2017-2018.

Sex	Age groups (Years)	Water	Milk	Coffee and tea	Fruit juices
		mL	mL	mL	mL
**Male**	**20-29**	2276 (2157, 2395)	29 (23, 36)	272 (254, 290)	268 (243, 292)
	**30-39**	2355 (2250, 2461)	21 (17, 26)	332 (314, 349)	242 (223, 261)
	**40-49**	2413 (2304, 2522)	23 (17, 29)	350 (325, 374)	246 (223, 268)
	**50-59**	2382 (2282, 2483)	31 (21, 41)	370 (349, 391)	205 (185, 226)
	**60-69**	2106 (1998, 2214)	42 (33, 51)	369 (348, 389)	174 (156, 191)
	**≥70**	1820 (1720, 1919)	56 (45, 68)	390 (357, 424)	169 (149, 191)
	**All men**	2280 (2226, 2333)	30 (27, 34)	338 (327, 348)	228 (218, 238)
**Female**	**20-29**	1892 (1803, 1981)	26 (21, 31)	248 (233, 262)	252 (231, 273)
	**30-39**	2005 (1923, 2087)	26 (21, 32)	305 (288, 322)	223 (208, 237)
	**40-49**	1988 (1913, 2063)	28 (22, 33)	326 (308, 345)	190 (176, 204)
	**50-59**	2006 (1929, 2083)	29 (24, 34)	358 (338, 378)	189 (175, 204)
	**60-69**	1948 (1860, 2035)	43 (34, 53)	380 (352, 408)	172 (155, 189)
	**≥70**	1685 (1589, 1781)	75 (61, 89)	359 (336, 382)	156 (137, 175)
	**All women**	1940 (1899, 1981)	34 (31, 37)	322 (313, 331)	203 (195, 211)
**Overall**		2100 (2058, 2141)	32 (30, 65)	329 (321, 338)	215 (207, 222)

Table S8. Estimate of the mean change in calorie intake (kcal/person/day) in the 3 taxation scenarios for all sugary beverages with a tax pass-through rate of 80%, according to sex and age groups.

**Sex**	**Age groups (years)**	**20% Tax** Mean (95% CI)	**25% Tax** Mean (95% CI)	**30% Tax** Mean (95% CI)
**Male**	**20 to 29**	-2.5 (-4.9;-0.2)	-3.2 (-6.1;-0.3)	-3.8 (-7.3;-0.4)
	**30 to 39**	3.0 (1.0;5.0)	3.8 (1.2;6.3)	4.5 (1.5;7.5)
	**40 to 49**	7.8 (6.2;9.4)	9.7 (7.8;11.7)	11.7 (9.3;14.0)
	**50 to 59**	10.2 (8.6;11.7)	12.7 (10.8;14.7)	15.3 (13.0;17.5)
	**60 to 69**	10.9 (9.3;12.5)	13.6 (11.6;15.6)	16.3 (14.0;18.7)
	**≥70**	12.1 (9.5;14.8)	15.2 (11.9;18.5)	18.2 (14.2;22.1)
	**All men**	5.6 (4.7;6.5)	7.0 (5.9;8.2)	8.4 (7.1;9.8)
**Female**	**20 to 29**	0.5 (-1.2;2.3)	0.7 (-1.5;2.8)	0.8 (-1.8;3.4)
	**30 to 39**	6.8 (5.5;8.1)	8.5 (6.9;10.1)	10.2 (8.2;12.1)
	**40 to 49**	8.8 (7.6;10.0)	10.9 (9.5;12.4)	13.1 (11.4;14.9)
	**50 to 59**	9.8 (8.4;11.2)	12.2 (10.5;14.0)	14.7 (12.6;16.8)
	**60 to 69**	12.7 (10.8;14.5)	15.8 (13.5;18.2)	19.0 (16.2;21.8)
	**≥70**	14.7 (13.1;16.3)	18.3 (16.3;20.3)	22.0 (19.6;24.4)
	**All women**	8.0 (7.3;8.7)	10.0 (9.2;10.8)	12.0 (11.0;13.0)
**Overall**		6.9 (6.2;7.5)	8.6 (7.8;9.4)	10.3 (9.4;11.3)

Table S9 – Estimate of the mean variation of BMI (kg/m^2) in the 3 taxation scenarios, according to sex and age groups.

		**Mean change in BMI in kg/m^2 (95% CI)**		
**Sex**	**Age groups**	**Tax 20%**	**Tax 25%**	**Tax 30%**
**Male**	**20-29**	-0.035 (-0.035;-0.035)	-0.044 (-0.045;-0.044)	-0.053 (-0.053;-0.053)
	**30-39**	0.042 (0.042;0.042)	0.053 (0.053;0.053)	0.063 (0.062;0.063)
	**40-49**	0.111 (0.110;0.111)	0.138 (0.137;0.138)	0.166 (0.165;0.167)
	**50-59**	0.146 (0.146;0.147)	0.182 (0.182;0.183)	0.219 (0.219;0.220)
	**60-69**	0.159 (0.159;0.160)	0.199 (0.198;0.200)	0.238 (0.238;0.239)
	**≥70**	0.179 (0.179;0.180)	0.225 (0.224;0.226)	0.270 (0.269;0.271)
	**All men**	**0.084 (0.083;0.086)**	**0.105 (0.103;0.107)**	**0.126 (0.124;0.129)**
**Female**	**20-29**	0.008 (0.008;0.008)	0.011 (0.011;0.011)	0.013 (0.013;0.013)
	**30-39**	0.110 (0.110;0.111)	0.138 (0.137;0.138)	0.165 (0.165;0.166)
	**40-49**	0.144 (0.143;0.144)	0.178 (0.178;0.179)	0.214 (0.214;0.215)
	**50-59**	0.162 (0.161;0.163)	0.202 (0.201;0.202)	0.243 (0.242;0.244)
	**60-69**	0.213 (0.212;0.214)	0.265 (0.264;0.266)	0.319 (0.318;0.320)
	**≥70**	0.253 (0.252;0.254)	0.315 (0.313;0.316)	0.378 (0.377;0.380)
	**All women**	**0.138 (0.137;0.139)**	**0.172 (0.170;0.173)**	**0.206 (0.204;0.208)**
**Overall**		**0.113 (0.111;0.114)**	**0.140 (0.139;0.142)**	**0.169 (0.167;0.170)**

Table S10. Percentage change in overweight and obesity prevalence (relative change in percent), by age and sex as a result of a 20, 25 and 30% SSB tax.

Sex	Age groups	Percentage change overweight (%)			Percentage change obesity (%)		
		Tax 20%	Tax 25%	Tax 30%	Tax 20%	Tax 25%	Tax 30%
**Male**	**20-29**	-1.88 (-1.97; -1.76)	-1.55 (-1.68; -1.39)	-1.55 (-1.68; -1.39)	-0.78 (-0.88; -0.71)	-2.00 (-2.18; -1.85)	-2.00 (-2.18; -1.85)
	**30-39**	0.21 (0.19; 0.23)	0.19 (0.17; 0.21)	0.21 (0.19; 0.23)	1.36 (1.26; 1.48)	1.39 (1.28; 1.51)	1.39 (1.28; 1.51)
	**40-49**	1.33 (1.31; 1.36)	1.58 (1.56; 1.60)	1.65 (1.64; 1.67)	1.73 (1.60; 1.88)	1.81 (1.68; 1.97)	1.90 (1.76; 2.08)
	**50-59**	2.87 (2.79; 2.95)	2.91 (2.85; 2.99)	4.55 (4.42; 4.71)	2.23 (2.17; 2.31)	2.68 (2.63; 2.74)	2.70 (2.65; 2.76)
	**60-69**	4.51 (4.28; 4.78)	6.00 (5.74; 6.30)	5.46 (5.18; 5.78)	1.41 (1.35; 1.48)	1.52 (1.46; 1.60)	3.30 (3.14; 3.49)
	**≥70**	6.23 (6.04; 6.38)	8.50 (8.36; 8.62)	6.43 (6.12; 6.71)	0.57 (0.51; 0.64)	0.57 (0.51; 0.64)	7.74 (7.11; 8.57)
	**All men**	1.64 (1.55; 1.73)	2.13 (2.04; 2.22)	2.21 (2.12; 2.31)	1.31 (1.26; 1.37)	1.29 (1.24; 1.35)	1.99 (1.90; 2.08)
**Female**	**20-29**	0.03 (0.03; 0.03)	0.25 (0.23; 0.26)	0.25 (0.23; 0.26)	0.08 (0.07; 0.09)	0.08 (0.07; 0.09)	0.08 (0.07; 0.09)
	**30-39**	2.23 (2.16; 2.31)	2.27 (2.20; 2.35)	2.76 (2.66; 2.86)	2.09 (1.96; 2.26)	2.26 (2.11; 2.44)	2.41 (2.26; 2.60)
	**40-49**	2.78 (2.36; 3.15)	3.56 (3.18; 3.89)	3.53 (3.15; 3.87)	1.46 (1.37; 1.58)	1.65 (1.54; 1.78)	2.02 (1.89; 2.18)
	**50-59**	3.06 (2.93; 3.20)	3.26 (3.13; 3.40)	5.36 (5.21; 5.53)	2.77 (2.69; 2.87)	3.00 (2.91; 3.10)	3.12 (3.02; 3.23)
	**60-69**	4.13 (4.05; 4.23)	4.63 (4.52; 4.77)	3.08 (3.00; 3.16)	3.41 (3.27; 3.58)	3.95 (3.79; 4.14)	7.79 (7.47; 8.16)
	**≥70**	6.13 (5.90; 6.39)	6.53 (6.40; 6.66)	5.76 (5.66; 5.88)	2.65 (2.47; 2.86)	4.55 (4.36; 4.78)	7.32 (6.97; 7.75)
	**All women**	2.90 (2.89; 2.91)	3.26 (3.25; 3.27)	3.43 (3.41; 3.45)	2.10 (2.04; 2.17)	2.48 (2.41; 2.56)	3.45 (3.35; 3.56)
**Overall**		2.25 (2.22; 2.28)	2.68 (2.65; 2.71)	2.80 (2.78; 2.83)	1.76 (1.70; 1.82)	1.97 (1.91; 2.04)	2.82 (2.72; 2.93)
